# ﻿Review of the Palaearctic species of *Miscogasteriella* Girault, 1915 (Chalcidoidea, Pteromalidae)

**DOI:** 10.3897/zookeys.1154.101189

**Published:** 2023-03-20

**Authors:** Ekaterina V. Tselikh, Jaehyeon Lee, Deok-Seo Ku

**Affiliations:** 1 Zoological Institute, Russian Academy of Sciences, St Petersburg 199034, Russia Zoological Institute, Russian Academy of Sciences St Petersburg Russia; 2 The Science Museum of Natural Enemies, Geochang 50147, Republic of Korea The Science Museum of Natural Enemies Geochang Republic of Korea

**Keywords:** Description, key, new record, new species, parasitoid, Trigonoderinae

## Abstract

Palaearctic species of the genus *Miscogasteriella* Girault, 1915 are reviewed. *Miscogasteriellaolgae***sp. nov.** from South Korea and *M.vladimiri***sp. nov.** from Japan are described. Type material of *M.nigricans* (Masi) and *M.sulcata* (Kamijo) is redescribed and illustrated. *Miscogasteriellanigricans* is recorded from the Palaearctic region for the first time. An identification key to females of all Palaearctic species of *Miscogasteriella* is given.

## ﻿Introduction

The pteromalid genus *Miscogasteriella* Girault, 1915 (type species *Miscogasteriellalongiventris* Girault, 1915) belongs to the family Pteromalidae, subfamily Trigonoderinae (Burks et al. 2022), and is distributed in the Palaearctic, Oriental, and Australian regions. Until present, it comprised ten species, with only *Miscogasteriellasulcata* (Kamijo, 1962) being found in the Palaearctic region (Kamijo 1962; Tselikh et al. 2017; Noyes 2019).

Eight other species of *Miscogasteriella*, *M.bijoyi* Sureshan & Nihkil, 2013, *M.burmanica* (Hedqvist, 1968), *M.flavipes* (Masi, 1927), *M.jayasreeae* Sureshan, 1999, *M.keijli* Narendran, 2012, *M.nigricans* (Masi, 1927), *M.perakensis* (Hedqvist, 1968), *M.yemenica* Narendran & van Harten, 2007 are distributed in the Oriental region (Masi 1927; Hedqvist 1968; Sureshan 1999; Narendran and Harten 2007; Narendran 2012; Sureshan and Nikhil 2013; Noyes 2019).

Two species, *M.niger* (Bouček, 1988) and *M.longiventris* Girault, 1915, are distributed in the Australian region (Girault 1915; Bouček 1988; Noyes 2019).

Unfortunately, the biology is unknown for all species of *Miscogasteriella*, but mostly they were collected near dead trees in forests, suggesting similar hosts to other Trigonoderinae.

The aim of this work is to describe two new species of *Miscogasteriella* from South Korea and Japan, and to redescribe and illustrate the species *M.nigricans* and *M.sulcata*. An identification key to females of all Palaearctic species of *Miscogasteriella* is also provided.

## ﻿Materials and methods

The specimens examined in this study are deposited in the collections of the Deutsches Entomologisches Institut (Eberswalde, Germany; **DEI**), the Department of Life Sciences of the Yeungnam University (Gyeongsan, Republic of Korea; **YNU**), the National Institute of Biological Resources (Incheon, Republic of Korea; **NIBR**), the Science Museum of Natural Enemies (Geochang, Republic of Korea; **SMNE**), the Korea National Arboretum (Pocheon, Republic of Korea; **KNA**), the Entomological Laboratory of the Hokkaido University (Sapporo, Japan; **EIHU**), the Ehime University Museum (Matsuyama, Japan; **EUM**), and the Zoological Institute of the Russian Academy of Sciences (St Petersburg, Russia; **ZISP**).

Morphological terminology, including sculpture and wing venation, follows Bouček and Rasplus (1991), Gibson (1997), and Burks et al. (2022). The flagellum consists of two anelli, six funicular segments, and the four-segmented clava. The following abbreviations are used: **POL** – posterior ocellar line, the minimum distance between the posterior ocelli; **OOL** – ocello–ocular line, the minimum distance between a posterior ocellus and compound eye; **C1–C4** – claval segments; **PST** – parastigma; **M** – marginal vein; **S** – stigmal vein; **PM** – postmarginal vein; **F1–F6** – funicular segments; **Mt2–Mt8** – metasomal tergites (Mt1 – petiole). The scape is measured without the radicle; the pedicel is measured in lateral view. The distance between the clypeal lower margin and the toruli is measured from the lower margins of the toruli. Eye height is measured as the maximum diameter, eye length as the minimum diameter. The mesosoma and metasoma are measured in lateral view, the latter including the ovipositor sheaths.

Specimens were examined using Olympus SZX12, Nikon SMZ745T and Zeiss SteREO Discovery V20 stereomicroscopes. Photographs were taken with a Canon EOS 70D digital camera mounted on an Olympus SZX10 microscope (ZISP specimens), and a Digital Sight PS-Fi2 camera mounted on a Nikon SMZ745T microscope (EIHU specimens). The acquired images were then processed with Helicon Focus.

## ﻿Taxonomy

**Class Hexapoda Blainville, 1816**

**Order Hymenoptera Linnaeus, 1758**

**Family Pteromalidae Dalman, 1820**

**Subfamily Trigonoderinae Bouček, 1964**

### ﻿Genus Miscogasteriella

Taxon classificationAnimaliaHymenopteraPteromalidae

Girault, 1915

A643F3A0-3289-5D29-BEDC-099DAD3F3A1C

Miscogasteriella Girault, 1915: 196–197. Type species Miscogasteriellalongiventris Girault, 1915, by original designation.
Glyptosticha Masi, 1927: 348–349. Type species Glyptostichaflavipes Masi, 1927, by original designation. Subjective synonym of Miscogasteriella Girault, 1915 in Bouček (1988: 402).
Trigonoderoides Kamijo, 1962: 121–122. Type species Glyptostichanigricans Masi, 1927, by original designation and monotypy. Subjective synonym of Miscogasteriella Girault, 1915 in Heydon (1997: 5, 13, 73).

#### Diagnosis.

Vertex of head smooth (Figs 4, 13, 20, 28). Clypeal margin with angular median tooth (Fig. 3) or weakly emarginate (Figs 12, 19, 27); tentorial pits distinct, but shallow (Figs 3, 12, 19, 27); antennal formula 11264 female (Figs 2, 11, 22, 30) and 11210 male (Figs 17, 25, 33); scutellum with distinct frenal area (Figs 5, 14, 24, 31); propodeum with medial longitudinal depression (Figs 14, 24, 31) or median pit (Fig. 5); fore wing without speculum (Fig. 29) or in form of a narrow line near basal vein (Figs 7, 15, 21).

#### Distribution.

Palaearctic, Oriental and Australian regions.

### ﻿Key to Palaearctic species of *Miscogasteriella* based on females

**Table:** 

1	Lower margin of clypeus with angular median tooth (Fig. 3). Antennal scape extending to middle ocellus, 1.31–1.35 times as long as eye length. Propodeum with costula (Fig. 5). Fore wing with PST 1.15–1.17 times as long as M (Fig. 7)	***M.nigricans* (Masi, 1927)**
–	Lower margin of clypeus weakly emarginate (Figs 12, 19, 27). Antennal scape not extending to middle ocellus, 0.89–1.05 times as long as eye length. Propodeum without costula (Figs 14, 24, 31). Fore wing with PST 0.55–0.83 times as long as M (Figs 15, 21, 29)	**2**
2	F1 with 5–6 rows of sensilla (Fig. 22). Fore wing with PST 0.76–0.83 times as long as M (Fig. 21). Frenum with finely reticulate sculpture (Fig. 24). Propodeal lateral depressions 0.55–0.60 times as long as propodeum (Fig. 24)	***M.sulcata* (Kamijo, 1963)**
–	F1 with 3–4 rows of sensilla (Figs 11, 30). Fore wing with PST 0.55–0.67 times as long as M (Figs 15, 29). Frenum with alutaceous sculpture (Figs 14, 31). Propodeal lateral depressions 0.30–0.44 times as long as propodeum (Figs 14, 31)	**3**
3	Combined length of pedicel and flagellum 1.75 times breadth of head. Dorsellum with distinct upper crenulate cross-line (Fig. 14). Scape (Figs 11, 12) and femora brown (Fig. 10). Metasoma with Mt2-Mt4 metallic blue-green with diffuse coppery lustre, Mt5–Mt8 brown with diffuse violet-coppery lustre (Fig. 16)	***M.olgae* sp. nov.**
–	Combined length of pedicel and flagellum 1.56–1.57 times breadth of head. Dorsellum without distinct upper crenulate cross-line (Fig. 31). Scape (Figs 27, 30) and femora yellowish-brown (Fig. 26); metasoma cupreous (Fig. 32)	***M.vladimiri* sp. nov.**

### ﻿ Miscogasteriella nigricans

Taxon classificationAnimaliaHymenopteraPteromalidae

(Masi, 1927)

71491210-E14A-534A-9DB2-7D20897E3930

Figs 1–8

Glyptosticha nigricans Masi, 1927: 353. Syntype female (DEI, examined) recognised by Masi, 1927: 353.

#### Type material.

***Syntype***: female, “Taiwan, Hoozan Formosa H. Sauter, 1910”, “TYPUS”, “Dtsch. Entomol. Institut Berlin”, “*Glyptostichanigricans* ♀ Masi”, “Coll. DEI Eberswalde”, “*Trigonoderoidesnigricans* (Masi) Det. K. Kamijo”, “GBIF-ChalciSD ID: ChalD0419” (DEI).

**Figures 1–8.**
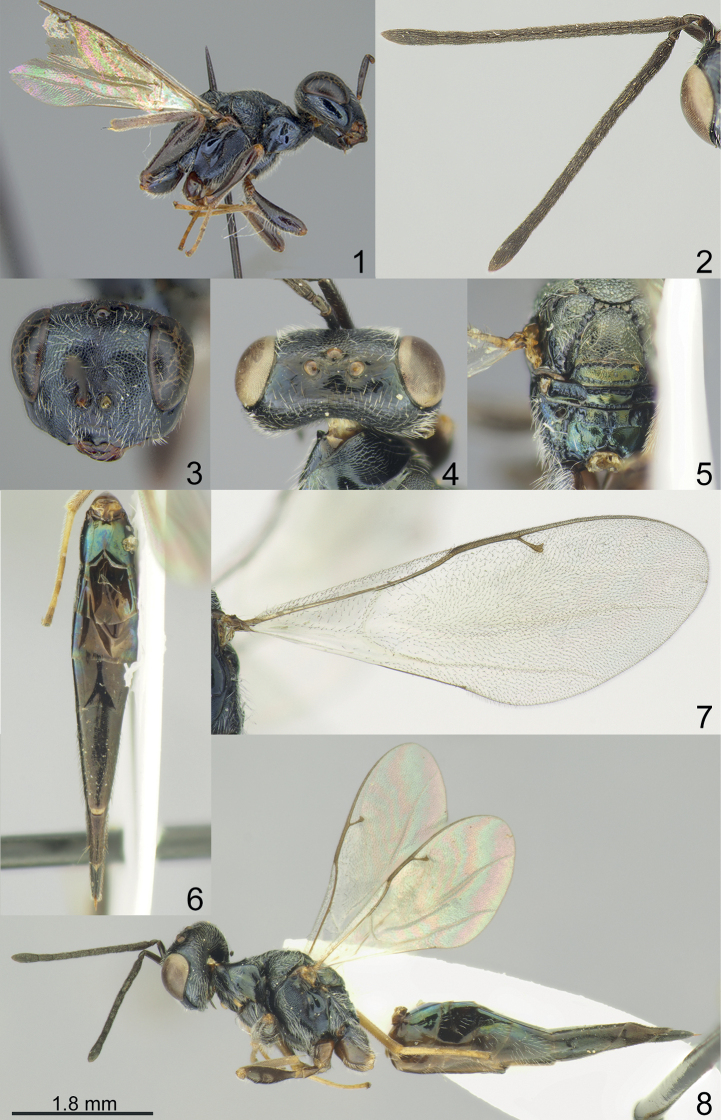
**1, 3***Miscogasteriellanigricans* (Masi, 1927), female, syntype **2, 4–8** female, non-type **1** head and mesosoma, lateral view **2** antenna **3** head, frontal view **4** head, dorsal view **5** scutellum and propodeum, dorsal view **6** metasoma, dorsal view **7** fore wing **8** habitus, lateral view.

#### Additional material examined.

Russia: 1 female, “Primorskii Reg., 40 km NE Spassk-Dalny Town, Dukhovskoe Vill, 1.VIII.1996, S. Belokobylskij” (ZISP).

#### Description.

**Female.** Body length 7.70–9.60 mm; fore wing length 4.40 mm (wings of syntype are broken).

***Coloration*.** Head black. Antenna with scape, pedicel, and flagellum dark brown. Mesosoma black, pronotum and mesoscutum dorsally with metallic diffuse green-coppery lustre, propodeum dorsally with metallic diffuse blue-coppery lustre. All coxae and all femora brown; tibiae and tarsi yellowish-brown. Fore wing slightly infuscate, venation yellowish-brown. Metasoma dark brown, in dorsal view Mt2-Mt4 metallic blue-green with diffuse coppery lustre.

***Sculpture*.** Head in frontal view reticulate, head in dorsal view and clypeus smooth and shiny; mesosoma reticulate, but frenum finely reticulate; dorsellum alutaceous, with distinct upper and lower crenulate cross-line; propodeum weakly reticulate; metasoma weakly alutaceous and shiny.

***Head*.** Head in dorsal view 2.23–2.25 times as broad as long and 1.20–1.21 times as broad as mesoscutum; in frontal view 1.26–1.29 times as broad as high. POL 0.90–0.91 times as long as OOL. Eye height 1.40–1.50 times eye length and 2.80–3.00 times as long as malar space. Distance between antennal toruli and lower margin of clypeus 0.55–0.60 times distance between antennal toruli and median ocellus. Lower margin of clypeus with angular median tooth. Antenna with scape 0.87–0.90 times as long as eye height and 1.31–1.35 times as long as eye length; pedicel 1.80–2.00 times as long as broad and 0.25–0.37 times as long as F1; combined length of pedicel and flagellum 1.67–1.70 times breadth of head; F1 3.80–4.00 times as long as broad and with 4–5 rows of sensilla, F3–F6 longer than broad; clava 3.07–3.40 times as long as broad, with micropilosity area on C3, and part of C2.

***Mesosoma*.** Mesosoma 1.80–1.95 times as long as broad. Scutellum 1.06–1.10 times as long as broad. Propodeum without nucha, with costula, 0.59–0.60 times as long as scutellum. Fore wing 2.65 times as long as maximum width; basal cell, cubital vein, basal vein pilose; speculum as narrow line near basal vein; PST 1.15–1.17 times as long as M, M 0.52–0.53 times as long as P and 2.67–2.80 times as long as S.

***Metasoma*.** Metasoma 5.20–5.55 times as long as broad, 1.96–2.05 times as long as mesosoma and 1.42–1.43 times as long as mesosoma and head; Mt8 2.25–2.40 times as long as broad.

**Male.** Unknown.

#### Distribution.

Russian Far East, Taiwan.

### ﻿ Miscogasteriella olgae sp. nov.

Taxon classificationAnimaliaHymenopteraPteromalidae

EB927BC0-4D55-5145-A0BA-DF420B180E24

https://zoobank.org/ECA92916-6803-436B-984B-96DFA7DE2985

Figs 9–17

#### Type material.

***Holotype***: female, South Korea: “Korea, Gyeongsangnam-do, Goseong-gun, Hail-myeon, Suyang-ri, 34°58'35"N, 128°12'08"E, 18.VI.2022, E. Tselikh” (NIBR). ***Paratypes***: 3 males, same data as holotype (1 specimen in NIBR, 2 specimens in SMNE); 1 male, “Korea, Gyeongsangnam-do, Namhae-gun, Gohyeon-myeon, Daegok-ri, Hwabangsa temple, 34°51'07"N, 127°51'31"E, 19.VI.2022, E. Tselikh” (ZISP).

**Figures 9–17.**
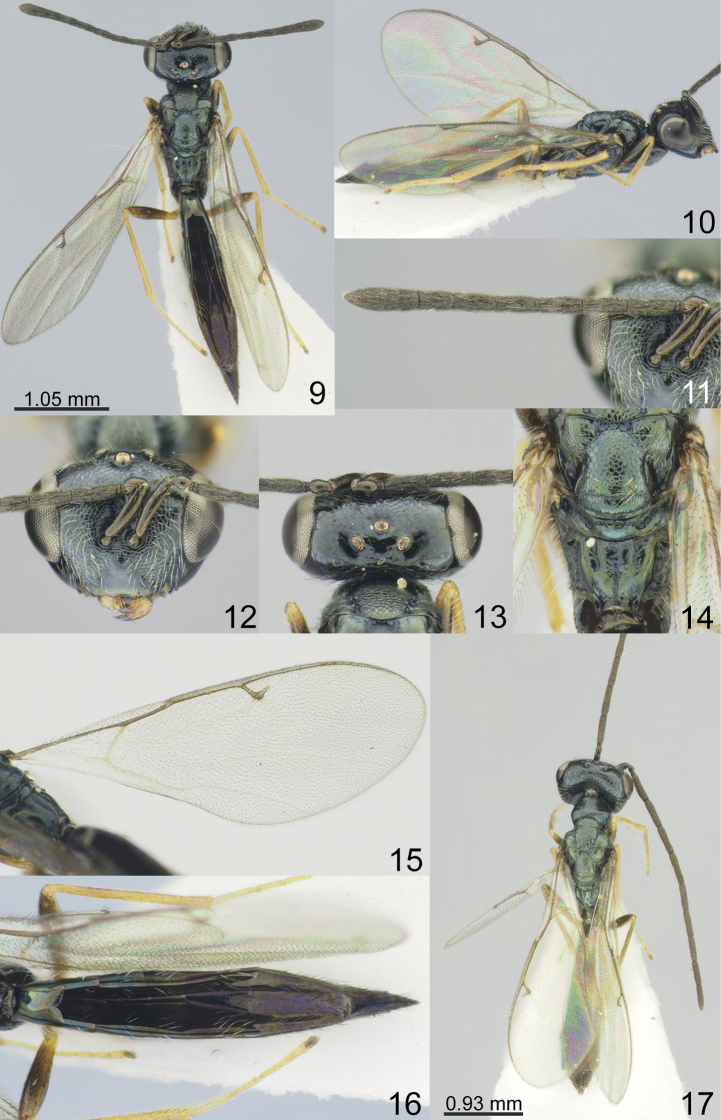
**1–16***Miscogasteriellaolgae* sp. nov., female, holotype **17** male, paratype **9** habitus, dorsal view **10** habitus, lateral view **11** antenna **12** head, frontal view **13** head, dorsal view **14** scutellum and propodeum, dorsal view **15** fore wing **16** metasoma, dorsal view **17** habitus, dorsal view.

#### Description.

**Female.** Body length 4.20 mm; fore wing length 3.20 mm.

***Coloration*.** Head black, dorsally with metallic blue lustre. Antenna with scape, pedicel, and flagellum brown. Mesosoma metallic blue-green with diffuse coppery lustre. All coxae brown with metallic blue lustre; all femora brown; tibiae, and tarsi yellow. Fore wing hyaline, venation yellowish-brown. Metasoma with Mt2-Mt4 metallic blue-green with diffuse coppery lustre, Mt5–Mt8 brown with diffuse violet-coppery lustre.

***Sculpture*.** Head in frontal view reticulate, head in dorsal view and clypeus smooth and shiny; mesosoma reticulate, but axilla and frenum alutaceous; dorsellum weakly alutaceous, with distinct upper crenulate cross-line, and without lower crenulate cross-line; propodeum weakly alutaceous; metasoma weakly alutaceous and shiny.

***Head*.** Head in dorsal view 2.30 times as broad as long and 1.65 times as broad as mesoscutum; in frontal view 1.30 times as broad as high. POL 0.80 times as long as OOL. Eye height 1.33 times eye length and 3.10 times as long as malar space. Distance between antennal toruli and lower margin of clypeus 0.78 times distance between antennal toruli and median ocellus. Lower margin of clypeus weakly emarginate. Antenna with scape 0.68 times as long as eye height and 0.90 times as long as eye length; pedicel 1.28 times as long as broad and 0.42 times as long as F1; combined length of pedicel and flagellum 1.75 times breadth of head; F1 3.00 times as long as broad and with 3–4 rows of sensilla, F3–F6 longer than broad; clava 3.05 times as long as broad, with micropilosity area on C3 and C2.

***Mesosoma*.** Mesosoma 2.00 times as long as broad. Scutellum 1.05 times as long as broad. Propodeum without nucha and costula, 0.78 times as long as scutellum; medial longitudinal depression shallow, lateral depressions 0.44 times as long as propodeum. Fore wing 2.80 times as long as maximum width; basal cell, cubital vein, basal vein pilose; speculum absent; PST 0.55 times as long as M, M 0.53 times as long as P and 3.60 times as long as S.

***Metasoma*.** Metasoma 6.20 times as long as broad, 2.03 times as long as mesosoma and 1.37 times as long as mesosoma and head; Mt8 1.67 times as long as broad.

**Male.** Body length 3.50–3.70 mm; fore wing length 2.80–3.00 mm. Head in frontal view 1.24–1.25 times as broad as high. Distance between antennal toruli and lower margin of clypeus 0.90–0.93 times distance between antennal toruli and median ocellus. Antenna with scape 0.48–0.52 times as long as eye height and 0.68–0.70 times as long as eye length. Combined length of pedicel and flagellum 1.70–1.73 times breadth of head. Antennal formula 11210. Fore wing with M 3.78–3.80 times as long as S. Metasoma 5.00–5.15 times as long as broad, 1.68–1.70 times as long as mesosoma and 1.25–1.27 times as long as mesosoma and head. Otherwise, similar to female.

#### Etymology.

The species is named in honour of the senior author’s mother, Olga Tselikh.

#### Distribution.

Korean Peninsula.

### ﻿ Miscogasteriella sulcata

Taxon classificationAnimaliaHymenopteraPteromalidae

(Kamijo, 1962)

C092E791-C1F9-562E-A610-B47993301211

Figs 18–25

Glyptosticha sulcata Kamijo, 1962: 118. Holotype female (EIHU, examined) designated by Kamijo, 1962: 118.

#### Type material.

***Holotype***: female, Japan: “Japan, Kyushu, 11.VIII.1955, K. Nohara”, “Holotype *Glyptostichasulcata* Kamijo ♀” (EIHU).

**Figures 18–25.**
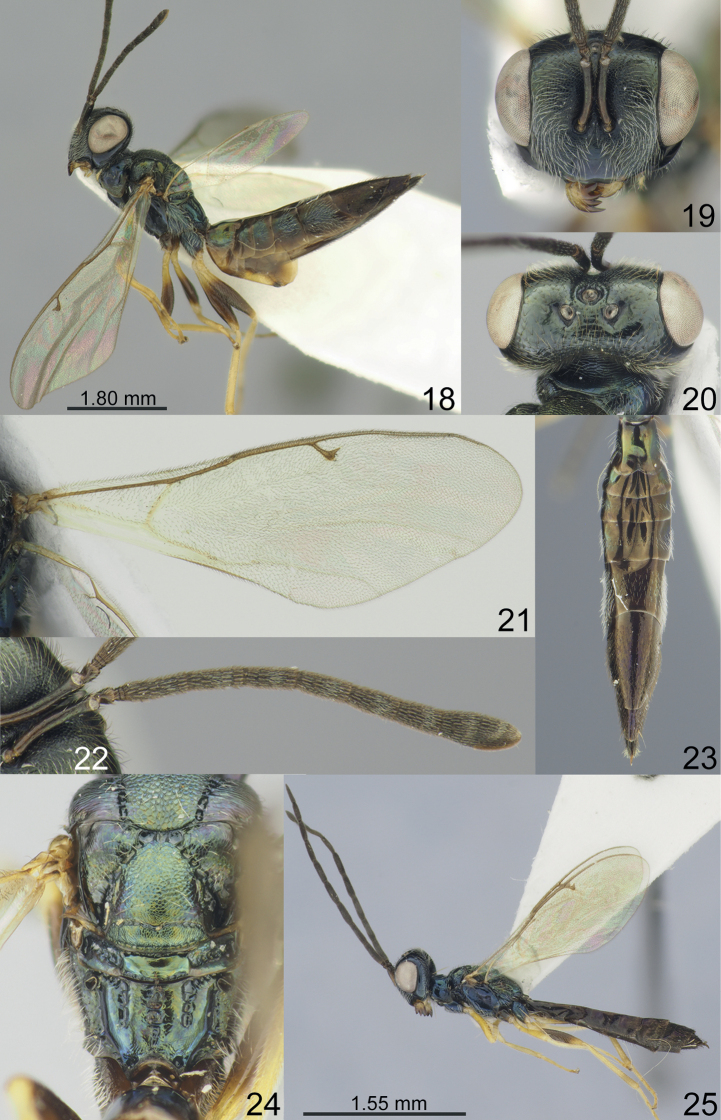
**18–24***Miscogasteriellasulcata* (Kamijo, 1963), female, non-type **25** male, non-type **18** habitus, lateral view **19** head, frontal view **20** head, dorsal view **21** fore wing **22** antenna **23** metasoma, dorsal view **24** scutellum and propodeum, dorsal view **25** habitus, lateral view.

#### Additional material examined.

South Korea: 2 females, “Busan, Gijang-gun, Jeonggwanmyeon, Gijang-cheongsonyeon-suryeonwon, 35°18'52"N, 129°09'57"E, 22.V–29.VI.2008, J.W. Lee”; 1 female, “Chungcheongbuk-do, Yeongdong-gun, Sangchon-myeon, Mulhan Valley, 35°49'53"N, 128°32'28"E, 23.V.2002, J.W. Lee”; 1 female “Miwon-ri, Miwon-myeon, Cheongwon-gun, 15–22.VII.05, J.H. Han”; 1 female, “Danyang-gun, Cheondong-ri, Mt. Sobaek, Temp. Bukbusa, 7.VII–2.VIII.2005, J.W. Lee”; 3 males, “Cheongwon-gun, Miwonmyeon, Miwon-ri, 9–16.IX.2005, J.H. Han” (all in YNU); 1 male, “Gyeongsangbuk-do, Kyeongju-si, Hyeongok-myeon, Namsa-ri, 2–09.IX.2015, J.T. Mun”; 1 female, “Cheongdo-gun, Unmun-myeon, Mt. Unmun, 35°38'09"N, 128°59'18"E, 23.V.2008, J.W. Lee”; 2 females, same locality, 15–29.IX.2005, J.O. Lim; 1 female, same locality, 23.V.2008, J.W. Lee; 2 females, same locality, 5–15.V.2009, J.W. Lee; 2 females, same locality, 30.V–16.VI.2009, C.J. Kim; 2 females, “Yeongju-si, Punggieup, Jungnyeong, 35°53'43"N, 128°26'22"E, 20.VIII–5.IX.2009, J.W. Lee”; 1 female, “Cheongdo-gun, Unmun-myeon, Haksodaepakpo, 35°38'15"N, 128°59'51"E, 2.VII–16.VIII.2013, J.W. Lee”; 2 females, “Cheongdo-gun, Unmun-myeon, Ssalbawi, Mt. Unmun, 35°38'08"N, 129°01'27"E, 13.VII–16.VIII.2013, J.W. Lee”; 6 males, “Chilgok-gun, Dongmyeongmyeon, Hakmyeong-ri, San, 36°01'53"N, 128°33'47"E, 15.VII–29.VIII.2014, J.W. Lee”; 1 female, “Yeongcheon-si, Sinnyeong-myeon, Chisan-ri, San 141-4, 36°01'13"N, 128°42'26"E, 15.VII–29.VIII.2014, J.W. Lee”; 1 female, “Yeongcheon-si, Sinnyeong-myeon, Chisan-ri, San141-4, 36°01'13"N, 128°42'26"E, 30.VIII–25.IX.2014, J.W. Lee”; 1 female, “Bonghwa-gun, Myeongho-myeon, Gwanchang-ri, Mt. Cheongryang, 22.V.2015, J.W. Lee”; 1 female, “Bonghwa-gun, Jaesan-myeon, Galsan-ri, Irwolsan-gil, 36°49'22"N, 129°05'05"E, 7.VIII.2015, E.V. Tselikh, K.H. Ko” (all in YNU); 2 females, “Daehyeon-ri, Bukhu-myeon, Andong-si, 31.V–16.VI. 2021, 15.X–5. XI. 2021, Malaise Trap, K. Gimyeon”; 3 males, “Daedong-ri, Mari-myeon, Geochang-gun, 15.VIII–25.VIII.2021, 8.IX–23.IX.2021, Malaise Trap, L. Jaehyeon, J. Hyojin” (all in SMNE); 2 males, “Gyeonggi-do, Anyang-si, Manan-gu, Gwanak Arboretum, 37°18'05"N, 127°19'02"E, 5–19.VII.2007, J.O. Lim”; 3 females, 1 male, “Gapyeong-gun, Cheongpyeong-myeon, Mt. Homyeong, 1–26.V.2009, J.W. Lee”; 3 females, 2 males, same locality, 37°43'16"N, 127°19'23"E, 31.VII–17.VIII.2009, 27.V–10.VI.2009, J.O. Lim; 1 male, “Namyangju-si, Choan-myeon, Songchon-ri, Mt. Ungil, 37°34'43"N, 127°18'38"E, 26.VI–16.VII.2009, J.O. Lim”; 1 female, 22 males, same locality, 37°34'43"N, 127°18'40"E, 18.VIII–4.IX.2009, J.O. Lim; 2 females, 1 male, “Pocheon-si, Soheur-eup, Jikdong-ri, 51-7, Korea National Arboretum, 37°45'02"N,127°08'34"E, 29.VIII–14.X.2013, I.G. Kim” (all in YNU); 3 females, 5 males, “Soheul-eup, Pocheon-si, 24.V–12.VI.2017, 30.VI–17.VII.2017, 31.VI–16.VIII.2017, 37°45'02"N, 127°08'35"E, Kim, Kim, Nam” (all in KNA); 5 males, “Gyeongsangnam-do, Namhae-gun, Namhae-eup, Asan-ri, 34°51'07"N, 127°51'31"E, 19.VI.2022, S. Belokobylskij, E. Tselikh” (all in ZISP); 1 male, “Gangwon-do, Donghae-si, Samhwa-dong, Mureunggyegok, 2–10.X.2006, J.W. Lee”; 1 female, 1 male, “Wonju-si, Heungeop-myeon, Maeji-ri, 234,Yonsei University, 21.V–27.VI.2014, H.Y. Han” (all in YNU); 1 male, “Mandae-ri, Haean-myeon, Yuggu-gun, 30.VI.2014, H.T. Shin” (in KNA). Japan: 1 female, “Shikoku, Kochi Pref., Ashizuri-misaki, 23.V.1983, M. Miyatake”; 1 female “Nagano Pref., Ueda City, Sugadaira-kougen, Tsukuba Univ., 36-31N/138-20E (about 1300 m), 9.IX.2013, S. Shimizu”; 1 male, “Niigata Pref., Nagaoka City, Suyoshi Town, Mt. Nokogiri-yama, (about 690 m), 21.VII.2014, S. Shimizu, R. Shimizu” (all in EUM).

#### Description.

**Female.** Body length 6.90–8.00 mm; fore wing length 4.90–5.60 mm.

***Coloration*.** Head dark blue, in frontal view metallic diffuse coppery green lustre. Antenna with scape, pedicel, and flagellum dark brown. Mesosoma dark blue-green, in dorsal view with a diffuse coppery lustre, propodeum dorsally metallic blue and partly with coppery lustre. Fore and hind coxae dark blue, middle coxae dark brown; all femora apically yellow, basally brown; tibiae, and tarsi yellow. Fore wing slightly infuscate, venation yellowish-brown. Metasoma dark brown, in dorsal view Mt2 and Mt3-Mt4 laterally metallic blue-green with diffuse coppery lustre.

***Sculpture*.** Head in frontal view weakly reticulate, in dorsal view and clypeus smooth and shiny; mesosoma reticulate, but frenum finely reticulate; dorsellum shiny, with distinct upper and lower crenulate cross-line; propodeum weakly reticulate; propodeum smooth and weakly reticulate only near medial longitudinal depression; metasoma weakly alutaceous and shiny.

***Head*.** Head in dorsal view 2.20–2.27 times as broad as long and 1.44–1.46 times as broad as mesoscutum; in frontal view 1.29–1.40 times as broad as high. POL 0.70–0.72 times as long as OOL. Eye height 1.39–1.42 times eye length and 3.40–3.60 times as long as malar space. Distance between antennal toruli and lower margin of clypeus 0.79–0.82 times distance between antennal toruli and median ocellus. Lower margin of clypeus weakly emarginate. Antenna with scape 0.69–0.74 times as long as eye height and 0.94–1.05 times as long as eye length; pedicel 1.55–1.62 times as long as broad and 0.20–0.38 times as long as F1; combined length of pedicel and flagellum 1.53–1.78 times breadth of head; F1 3.30–4.00 times as long as broad and with 5–6 rows of sensilla, F3–F6 longer than broad; clava 2.67–2.87 times as long as broad, with micropilosity area on C3 and C2.

***Mesosoma*.** Mesosoma 1.78–2.00 times as long as broad. Scutellum 1.07–1.10 times as long as broad. Propodeum without nucha and costula; 0.83–0.85 times as long as scutellum; medial longitudinal depression strong, lateral depressions 0.55–0.60 times as long as propodeum. Fore wing 2.80–2.89 times as long as maximum width; basal cell, cubital vein, basal vein pilose; speculum absent; PST 0.76–0.83 times as long as M, M 0.50–0.56 times as long as P and 3.00–3.35 times as long as S.

***Metasoma*.** Metasoma 3.97–4.48 times as long as broad, 1.81–1.88 times as long as mesosoma and 1.36–1.38 times as long as mesosoma and head; Mt8 1.09–1.25 times as long as broad.

**Male.** Body length 3.40–6.20 mm; fore wing length 3.10–4.20 mm. All coxae dark blue; fore and middle femora yellow, hind femora brown. Eye height 1.25–1.30 times eye length and 2.70–3.00 times as long as malar space. Antennal formula 11210; scape 0.52–0.60 times as long as eye height and 0.69–0.75 times as long as eye length; pedicel as long as broad; combined length of pedicel and flagellum 2.80–3.15 times breadth of head; F1 4.80–5.00 times as long as broad. Fore wing with PST 0.61–0.69 times as long as M. Metasoma 1.00–1.28 times as long as mesosoma and head. Otherwise, similar to female.

#### Distribution.

Korean Peninsula, Japan.

### ﻿ Miscogasteriella vladimiri sp. nov.

Taxon classificationAnimaliaHymenopteraPteromalidae

9DD748E9-C319-523C-A928-40DBEAF71AA7

https://zoobank.org/FFB5BCA3-EE5E-45A8-A5AC-C3A3B4CD7441

Figs 26–33

#### Type material.

***Holotype***: female, “Japan, Shikoku Isl., Muroto, Tosa, 8.VI.1959, M. Miyatake” (ZISP). ***Paratype***: 1 female, “Japan, Shikoku Isl., Kuroson, Tosa, 30.IV.1956, M. Miyatake” (EUM).

**Figures 26–33.**
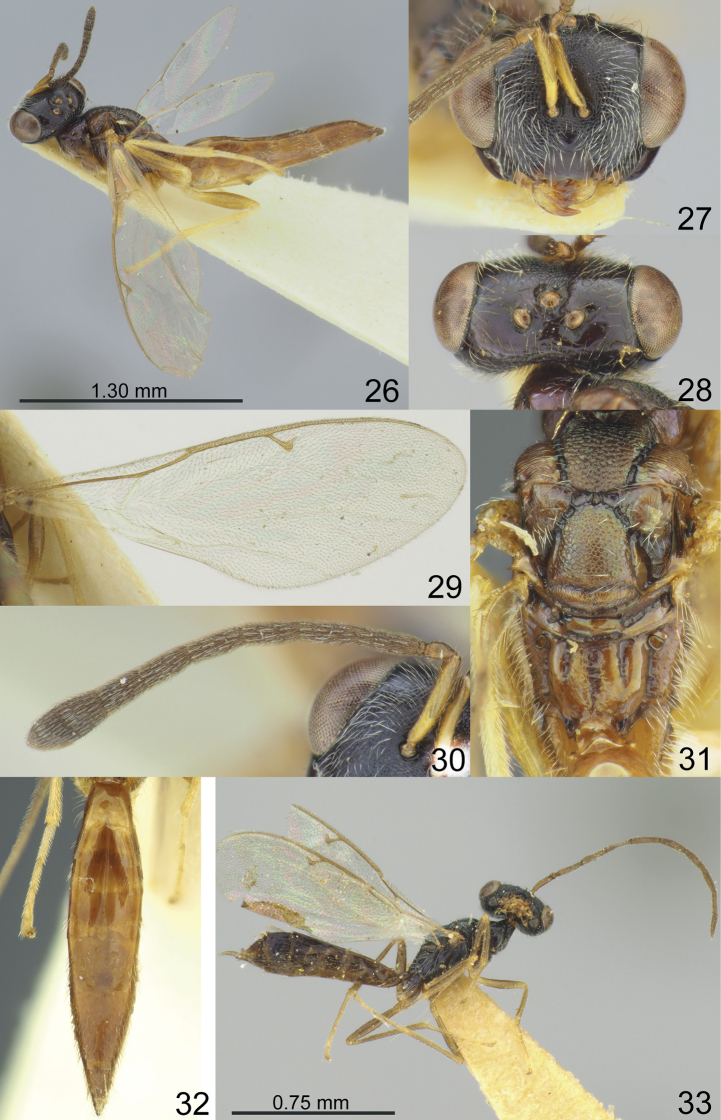
**26–32***Miscogasteriellavladimiri* sp. nov., female, holotype **33** male, paratype **26** habitus, lateral view **27** head, frontal view **28** head, dorsal view **29** fore wing **30** antenna **31** scutellum and propodeum, dorsal view **32** metasoma, dorsal view **33** habitus, lateral view.

#### Description.

**Female.** Body length 5.00–5.20 mm; fore wing length 3.90–4.10 mm.

***Coloration*.** Head dark brown. Antenna with scape yellowish-brown; pedicel, and flagellum brown. Mesosoma cupreous in lateral view with diffuse violet lustre. All coxae brown with diffuse violet lustre; all femora, tibiae, and tarsi yellowish-brown. Fore wing slightly infuscate; venation yellowish-brown. Metasoma cupreous.

***Sculpture*.** Head in frontal view weakly reticulate, in dorsal view and clypeus smooth and shiny; mesosoma reticulate, but axilla and frenum alutaceous; dorsellum shiny, without upper crenulate cross-line, and with lower crenulate cross-line; propodeum weakly reticulate; propodeum weakly reticulate propodeum weakly alutaceous; metasoma weakly alutaceous and shiny.

***Head*.** Head in dorsal view 2.10–2.17 times as broad as long and 1.66–1.69 times as broad as mesoscutum; in frontal view 1.30–1.34 times as broad as high. POL 0.77–0.80 times as long as OOL. Eye height 1.27–1.33 times eye length and 2.90–3.20 times as long as malar space. Distance between antennal toruli and lower margin of clypeus 0.85–0.97 times distance between antennal toruli and median ocellus. Lower margin of clypeus weakly emarginate. Antenna with scape 0.67–0.73 times as long as eye height and 0.89–0.90 times as long as eye length; pedicel 1.40–1.53 times as long as broad and 0.40–0.45 times as long as F1; combined length of pedicel and flagellum 1.56–1.57 times breadth of head; F1 2.95–3.00 times as long as broad and with 3 rows of sensilla, F3–F6 longer than broad; clava 2.30–2.50 times as long as broad, with micropilosity area on C3 and C2.

***Mesosoma*.** Mesosoma 2.00–2.10 times as long as broad. Scutellum 1.09–1.10 times as long as broad. Propodeum without nucha and costula, 0.85–1.00 times as long as scutellum; medial longitudinal depression shallow, lateral depressions 0.30–0.35 times as long as propodeum. Fore wing 2.79–2.82 times as long as maximum width; basal cell, cubital vein, basal vein pilose; speculum absent; PST 0.62–0.67 times as long as M, M 0.55–0.60 times as long as P and 3.40–3.44 times as long as S.

***Metasoma*.** Metasoma 4.00–4.65 times as long as broad, 1.77–1.95 times as long as mesosoma and 1.32–1.37 times as long as mesosoma and head; Mt8 1.00–1.10 times as long as broad.

**Male.** Body length 3.00 mm; fore wing length 2.60 mm. Eye height 1.13 times eye length and 3.40 times as long as malar space. Antennal formula 11210; scape 0.62 times as long as eye height and 0.70 times as long as eye length; pedicel 1.10 times as long as broad and 0.26 times as long as F1; combined length of pedicel and flagellum 1.78 times breadth of head; F1 4.67 times as long as broad and with 5 rows of sensilla. Mesosoma 2.00–2.10 times as long as broad. Scutellum 1.09–1.10 times as long as broad. Propodeum without nucha and transversal carina, 0.85–1.00 times as long as scutellum; medial longitudinal depression shallow, lateral depressions 0.30–035 times as long as propodeum. Fore wing 2.79–2.82 times as long as maximum width; basal cell, cubital vein, basal vein pilose; speculum absent; PST 0.62–0.67 times as long as M, M 0.55–0.60 times as long as P and 3.40–3.44 times as long as S. Metasoma 1.33 times as long as mesosoma and as long as mesosoma and head. Otherwise, similar to female.

#### Etymology.

The species is named in honour of the senior author’s father, Vladimir Tselikh.

#### Distribution.

Japan.

## Supplementary Material

XML Treatment for Miscogasteriella

XML Treatment for Miscogasteriella nigricans

XML Treatment for Miscogasteriella olgae

XML Treatment for Miscogasteriella sulcata

XML Treatment for Miscogasteriella vladimiri
